# Structural Abnormalities in Early Tourette Syndrome Children: A Combined Voxel-Based Morphometry and Tract-Based Spatial Statistics Study

**DOI:** 10.1371/journal.pone.0076105

**Published:** 2013-09-30

**Authors:** Yue Liu, Wen Miao, Jieqiong Wang, Peiyi Gao, Guangheng Yin, Liping Zhang, Chuankai Lv, Zhiying Ji, Tong Yu, B. A. Sabel, Huiguang He, Yun Peng

**Affiliations:** 1 Department of Radiology, Beijing Children’s Hospital, Capital Medical University, Beijing, China; 2 State Key Laboratory of Management and Control for Complex Systems, Institute of Automation, Chinese Academy of Sciences, Beijing, China; 3 Beijing key Lab of Magnetic Imaging Device and Technique, Beijing Children’s Hospital, Capital Medical University, Beijing, China; 4 Department of Neuroradiology, Beijing Tiantan Hospital, Capital Medical University, Beijing, China; 5 Medical Department, Beijing Children’s Hospital, Capital Medical University, West District, Beijing, China; 6 Otto-von-Guericke University of Magdeburg, Medical Faculty, Institute of Medical Psychology, Magdeburg, Germany; University of Texas Southwestern Medical Center, United States of America

## Abstract

Tourette Syndrome (TS) is characterized with chronic motor and vocal tics beginning in childhood. Abnormality of both gray (GM) and white matter (WM) has been observed in cortico-striato-thalamo-cortical circuits and sensory-motor cortex of adult TS patient. It is not clear if these morphological changes are also present in TS children and if there are any microstructural changes of WM. To understand the developmental cause of such changes, we investigated volumetric changes of GM and WM using VBM and microstructural changes of WM using DTI, and correlated these changes with tic severity and duration. T1 images and Diffusion Tensor Images (DTI) from 21 TS children were compared with 20 age and gender matched health control children using a 1.5T Philips scanner. All of the 21 TS children met the DSM-IV-TR criteria. T1 images were analyzed using DARTEL-VBM in conjunction with statistical parametric mapping (SPM). Diffusion tensor imaging (DTI) analysis was performed using Tract-Based Spatial Statistics (TBSS). Brain volume changes were found in left superior temporal gyrus, left and right paracentral gyrus, right precuneous cortex, right pre- and post- central gyrus, left temporal occipital fusiform cortex, right frontal pole, and left lingual gyrus. Significant axial diffusivity (AD) and mean diffusivity (MD) increases were found in anterior thalamic radiation, right cingulum bundle projecting to the cingulate gurus and forceps minor. Decreases in white matter volume (WMV) in the right frontal pole were inversely related with tic severity (YGTSS), and increases in AD and MD were positively correlated with tic severity and duration, respectively. These changes in TS children can be interpreted as signs of neural plasticity in response to the experiential demand. Our findings may suggest that the morphological and microstructural measurements from structural MRI and DTI can potentially be used as a biomarker of the pathophysiologic pattern of early TS children.

## Introduction

Tourette syndrome (TS) is a developmental neuropsychiatric disorder with the cardinal symptoms of motor and vocal tics which begin in childhood and fluctuate in severity in later years. The motor tics usually begin between the ages of 3-8 years, with the worst tic severity for most patients falls between age 7-15 year. Many patients may experience attenuation or remission of tic symptoms during adolescence. This may be due to drug intervention and/or patients’ improvement in their capacity for self-regulation of behavior [1]. TS is frequently concomitant with obsessive-compulsive disorder (OCD), attention-deficit hyperactivity disorder (ADHD), and other social and behavioral disturbances [2]. The incidence of TS is much higher than previously estimated [3], accounting for about 1% of children between the age 5–17 years [4,5] About 0.6% of the children also show symptoms of distress and impairment caused by their tics [6].

TS is typically diagnosed by observing symptoms and by evaluating the history of their onset. Thus far, only clinical measures, and not any brain morphological parameters, have been developed to diagnose TS. A prospective follow-up study of 43 TS children found that the volumes of caudate nucleus measured in childhood correlated significantly and inversely with the severity of tic and OCD symptoms in early adulthood [7], indicating the potential of MRI-based measures in clinical usage for TS.

Based on anatomical studies of post-mortem brains, it was proposed that TS arose from cortico-striato-thalamo-cortical (CSTC) circuit disturbances [8-10]. Specifically, reduced grey matter volumes were found in TS children in the basal ganglia [11] and increased in dorsal lateral prefrontal regions [12]. Morphological studies on TS also reported smaller corpus callosum volume [13] and thinning of sensorimotor cortices [14] in adults. Diffusion tensor imaging studies in adult TS patients found cerebral white matter fiber coherence changes underneath the sensorimotor cortex, the corticospinal tract and the genu of the corpus callosum [15-17]. However, there were only few publications on white matter changes in TS children [18,19].

Diffusion Tensor Imaging (DTI) is becoming widely used for its high sensitivity in detecting micro-structural alterations [20-22]. When analyzed with Tract-Based Spatial Statistics (TBSS) [23], DTI studies have the advantages of higher spatial registration and smoothing, thus enabling more accurate results. By using DTI, Neuner et al. [17] found a decrease of fractional anisotropy (FA) and an increase of radial diffusivity (RD) in the corticospinal tract and the anterior and posterior limb of the internal capsule in TS patients. Thomalla et al. [15] found FA increases in bilateral white matter underlying the post- and precentral gyrus and the right ventro-postero-lateral part of the thalamus. In addition to CSTC circuit involvement, disturbances in the anterior cingulate (limbic) cortex and the motor–cingulated-insular cortical neural system are also likely to play a critical role in TS pathology. The anterior cingulate (ACC) has numerous interconnections with the prefrontal and motor systems, other limbic regions, and the striatum -- regions that are suggested to be involved in TS pathology [24,25]. Structural and fMRI studies also showed gray matter alterations in motor loop as well as the limbic system [17,26], which may relate to the patient’s tics

Despite all of these findings, our understanding of the pathophysiological basis of TS is still limited. Adult TS patients may have comorbid ADHD and OCD, which often causes greater functional impairment to the individual than the tics. In fact, the suppression of tics may result in tension, and the patient may experience associated problems such as social isolation, embarrassment, and low self-esteem. Because these problems become only apparent in adulthood, it is unclear whether they are secondary consequences of the primary impairment or the results of brain damage earlier in life.

A clinically unbiased morphological and microstructural measure would be desirable to achieve TS prevention and design early intervention strategies. It is of great interest to quantify such measures in TS children, i.e. early in life [27]. In this study, we use DARTEL-VBM and TBSS to quantify grey and white matter morphological changes, to estimate the microstructural changes and to determine whether these changes are related to clinical measures such as YGTSS and duration of the disease. By studying TS children with short disease duration (1.84±0.56 yrs) who have no known ADHD or OCD, we were able to avoid confounding effects of medications and other diseases. We hypothesized that frontal cortices, limbic structures, and sensory-motor pathways may be altered in TS children, and these morphometric changes are associated with tic severity and duration.

## Materials and Methods

### Subjects

21 TS patients (age at imaging: 7.90±1.95 yrs; range: 5~11 yrs; 1 female) were recruited from outpatient clinics in our hospital from November 2009 to April 2010. All met DSM-IV-TR (Diagnostic and Statistical Manual of Mental Disorders, 4th Edition, text revision) criteria for TS. 20 age and gender matched health controls (age at imaging: 8.05±2.30 yrs; range: 4-10 yrs; 3 females) were included in this study. All controls were healthy, with no history of tic disorder, OCD, or ADHD. All participants were right-handed. Eight patients who had previously taken medications were drug-free for at least 1 month prior to entering the study [18]. The mean score of disease severity for all patients was 41.71±12.46 (range, 17.5-76.1) as documented with a Chinese translation of the Yale Global Tic Severity Scale (YGTSS) [28]. Clinical interview and the Children’s Yale-Brown Obsessive Compulsive Scale (CY-BOCS) [29] were used to diagnose OCD. The German short version of Wender Utah rating scale (WURS-k, translated to Chinese) [30] was used to diagnose ADHD. One TS patient had a history of uterine-incision delivery. The duration of TS ranged from 1 month to 5 years ([mean±STD]: 1.84±0.56 yrs). For those who had course less than 1 year, TS diagnosis was made by follow-up call. After the study was approved by Beijing Children’s Hospital review board, written informed consent was obtained from all the parents/guardians according to the Declaration of Helsinki. Details of the patients are shown in Table 1.

**Table 1:** Clinical details of the patients.

Subject	Age (yrs)	Sex	Duration (yrs)	YGTSS (0-100)	Tics overview
1	9	Male	1	40	Mmv-
2	10	Male	0.17	29	Mmv
3	9	Male	9	34	Mv-
4	6	Male	3.5	38	Mmv
5	5	Male	1.1	38	Mmv
6	10	Male	1.5	34	Mmv-
7	6	Male	0.5	39	Mmv-
8	8	Male	0.08	38	Mmv-
9	11	Male	1	39	Mmv-
10	7	Male	2	52	MmVv
11	7	Male	5	76	MmVv
12	5	Male	0.08	30	Mmv-
13	10	Male	1.1	42	Mmv
14	5	Male	1.1	56	MmVv
15	6	Male	1.1	53	MmVv
16	8	Male	0.08	17	Mmv-
17	9	Female	0.13	31	Mmv-
18	9	Male	4.5	39	MmVv
19	11	Male	4	44	Mmv-
20	7	Male	0.17	53	Mmv
21	8	Male	15	54	Mmv

Tics overview: m=simple motor, M=complex motor, v=simple vocal Tics, V=complex vocal Tics.

### Data acquisition

Magnetic resonance imaging was acquired using a 1.5T MR scanner (Gyroscan Interna Nova, Philips, Netherland). Head positioning was standardized using canthomeatal landmarks. After acquisition of a T2 localizer scan (TR = 1400ms, TE = 100ms, NEX3), axial three-dimensional T1-weighted image (3D T1WI) and diffusion tensor imaging (DTI) were acquired from all the subjects. 3D T1-weighted imaging were performed with axial three-dimensional-Fast Field Echo (3D FFE) sequence with the following parameters: repetition time (TR) = 25 ms, echo time (TE) = 4.6 ms, flip angle = 30°, reconstructed image matrix = 256×256, field of view (FOV) = 23×23 cm, slice thickness = 1 mm. DTI was performed using the following protocol: spin-echo diffusion-weighted echo-planar imaging sequence, 5 mm slice thickness, no inter-slice gap, repetition time = 4268 ms, echo time = 93 ms, field of view (FOV) = 23×23 cm, reconstructed image matrix = 192×192. Diffusion MRI images were obtained from 15 non-collinear directions with a b value of 1000 s/mm^2^.

### Quantification of local volumetric changes

After the data acquisition, 3D T1-weighted Images processing was performed by statistical parametric mapping (SPM8, http://www.fil.ion.ucl.ac.uk/spm, Wellcome Department of Cognitive Neurology, London, UK, 2008) and executed in Matlab 7.9 (MathWorks, Natick, MA, USA). All of the 3D T1-weighted images were reoriented with the origin set close to the anterior commissure (AC) and then were segmented into GM, WM and CSF in native space with unified segmentation [31]. Afterwards, all the segmented GM and WM images were rigidly transformed to produce a series of aligned GM and WM images. The study-specific GM/WM templates were then created with the aligned serial images from all the subjects and during the template creation processing, all aligned images were warped to the template yielding a series of flow fields, which parameterized the deformation. Data normalization was done, which was followed by data modulation to correct volume changes. Since the previous processing was performed in native space, it was necessary to transform the modulated data into MNI space. After the transformation, all the images were smoothed with a 6-mm full-width at half-maximum (FWHM) Gaussian filter. Two-sample t-test was then applied to evaluate the abnormalities of GM/WM between groups, using SPM8. Two contrasts were defined to examine both decreased and increased regions in the patients. Resulting statistical parametric maps of VBM were derived at a significance level of p < 0.001, uncorrected with an extent threshold of 10 voxels. Then small volume corrections (SVC) [32] limited to the volume of the regions we were interested in were performed using a sphere of 10mm radius.

### Quantification of microstructural changes

DTI images were transformed to nifti format in a workstation and processed off-line using FMRIB’s Diffusion Toolbox (FDT 2.0) within FSL 4.1 (*http://www.fmrib.ox.ac.uk/fsl*). For each subject, fifteen DTI volumes with b value of 1000 s/mm^2^ were first affine registered to the b0 volume for correction of eddy current distortion and simple head motion. Non-brain voxels were removed using Brain Extraction Tool (BET) [33] of FSL; a fractional intensity threshold of 0.3 was selected, resulting in a brain-extracted 4D image and a binary brain mask for each subject. The 4D image and corresponding brain mask created by BET were then used for fitting diffusion tensor model at each voxel using FDT. Eigenvalues of diffusion tensor matrix (λ_1_, λ_2_, λ_3_) were obtained and maps of axial diffusivity (AD, or λ_1_), mean diffusivity (MD = (λ_1_+λ_2_+λ_3_)/3), and fractional anisotropy (FA) were generated. Radial diffusivity (perpendicular eigenvalue, λ_23_ = (λ_2_+λ_3_)/2) was calculated by averaging the maps of λ_2_ and λ_3_. The standard TBSS [23,34] procedure was then applied to the data. First, all individual FA images were nonlinearly registered to the pre-defined FMRIB58_FA standard-space image provided by FSL, and affine-aligned into 1×1×1 mm MNI152 standard space. All subsequent processing was carried using this space and resolution. Then the mean of all FA images were created and fed into FA skeletonisation program to generate a FA skeleton, which represented the center of all tracts common to the subjects; a threshold of 0.2 was selected to define the borders of WM and GM. Each subject’s local maximum FA intensity along the perpendicular direction of white matter tract was projected to the mean FA skeleton to carry out the voxel-wise statistics across subjects. The same projection was applied to the MD, AD and RD images. Voxel-wise group comparisons of patients versus normal controls on the skeleton image were carried using FSL’s randomise tool. Randomise uses a permutation based statistical inference that does not rely on a Gaussian distribution [35]. Random Monte Carlo simulated samples of 10,000 permutations were used as null distribution. A statistical threshold p < 0.05, corrected for multiple comparisons with threshold-free cluster enhancement (TFCE) [36] method was used. TFCE can identify cluster-like structures without definition of an initial cluster-forming threshold or carrying out a large amount of data smoothing. The Johns Hopkins University JHU–ICBM-DTI-81 white-matter tractography atlas was used to identify the abnormal white matter tracts reveled by TBSS. TBSS results of MD, RD and AD were analyzed in the same manner.

### Link changes to clinical measures

To further determine the relation between the alterations and clinical scores and duration of tics, Pearson’s correlation was calculated using SPSS to relate corresponding values of that ROI (abnormal regions found by VBM) to the YGTSS scores, controlling for age, gender, and the whole brain volume. For DTI data, the identified tracks showed abnormal AD values were selected as ROI. The mean AD values in each ROI were correlated with the clinical data (YGTSS scores and duration of illness) using Pearson’s correlation, controlling for age and gender. The same operations were applied to the MD and FA results.

## Results

**Figure 1:**
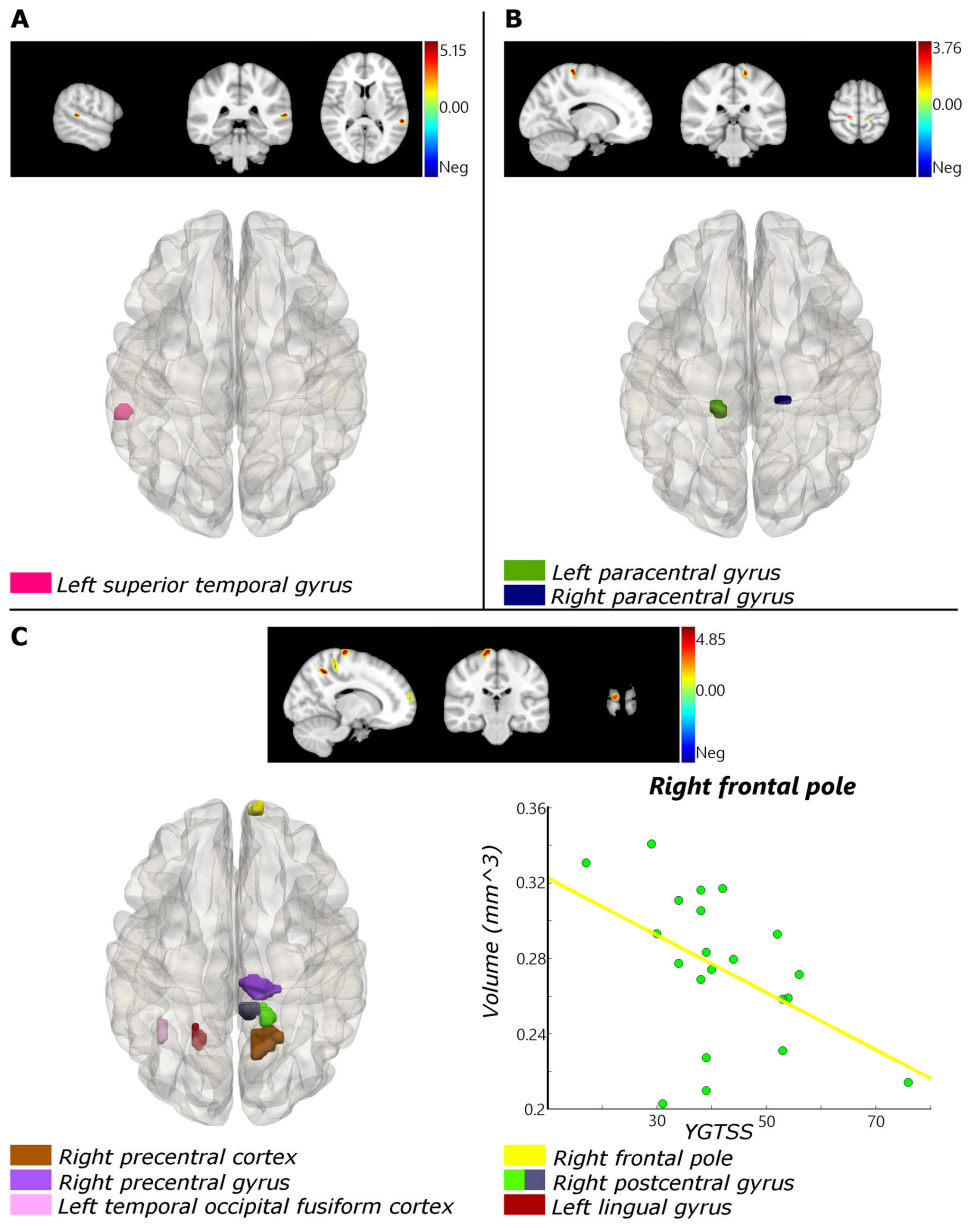
Brain areas with regional gray matter or white volume changes. (A) Reduced regional gray matter volumes (GMV) in left superior temporal gyrus. (B) Increased GMV in left and right precentral gyrus. (C) Reduced regional white matter volumes (WMV) in right precuneous cortex, right precentral gyrus, left temporal occipital fusiform cortex, right frontal pole, right postcentral gyrus and left lingual gyrus. Correlation between volume and YGTSS in right frontal pole (Lower right): r = -0.611, p = 0.005.

Compared with healthy controls, TS patients showed reduced regional gray matter volumes (GMV) in left superior temporal gyrus (Figure 1 A) and increased GMV in left and right precentral gyrus (Figure 1 B). WMV was decreased in right precuneous cortex, right precentral gyrus, left temporal occipital fusiform cortex, right frontal lobe, right postcentral gyrus and left lingual gyrus (Figure 1 C). No regions were found with increased WMV (Table 2).

**Table 2:** Brain areas with regional gray matter or white volume changes.

**Anatomical location (Brodmann area)**	**Cluster size**	**MNI-Space**			**t-value**	**p-value (SVC)**
		x	y	z		
**GMV: TS < Controls**						
Left superior temporal gyrus (42)	13	-58	-36	14	5.29	0.000
**GMV: TS > Controls**						
Left paracentral gyrus (6)	19	-12	-28	72	3.87	0.018
Right precentral gyrus (6)	10	18	-24	68	3.44	0.018
**WMV: TS < Controls**						
Right precuneous cortex (7)	71	16	-52	50	5.09	0.004
Right precentral gyrus (4)	55	14	-24	78	4.71	0.002
Left temporal occipital fusiform cortex (37)	11	-40	-56	-12	4.58	0.025
Right frontal pole (10)	19	12	64	14	4.53	0.018
Right postcentral gyrus (3)	20	16	-38	64	4.35	0.025
	24	10	-34	72	4.32	0.040
Left lingual gyrus (19)	21	-20	-56	2	3.54	0.017

MNI: Montreal Neurological Institute; GMV: gray matter volume; WMV: white matter volume

**Figure 2:**
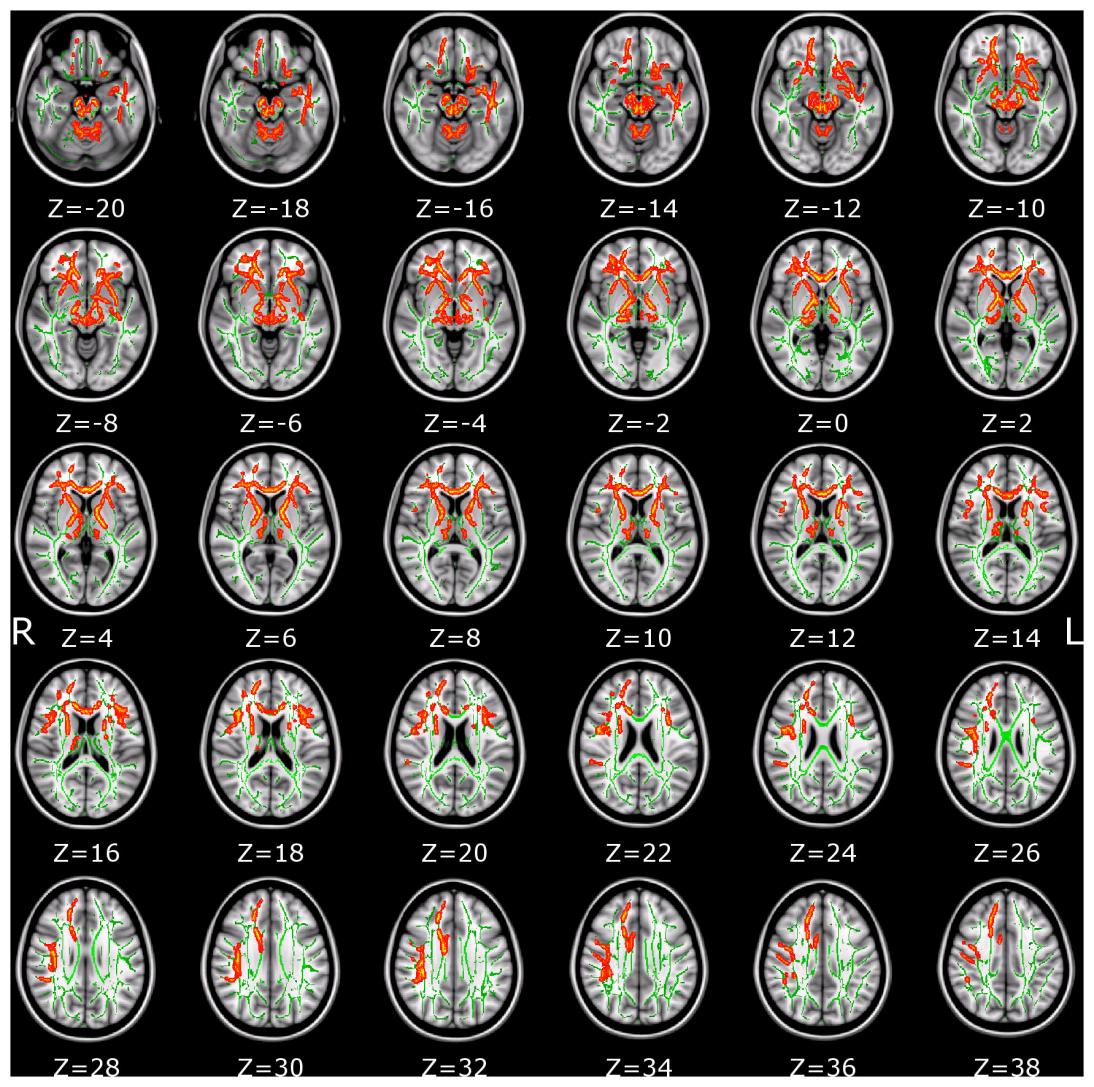
The identified AD skeleton clusters at p < 0.05 (corrected for multiple comparisons) were filled (using tbss_fill script implemented in FSL) to make the presentation easy. Changes can be found in different brain areas. The white-matter microstructure is altered in the anterior limb of the internal capsule, the corpus callosum and long association fibers such as the inferior longitudinal fascicle. The background image is the standard MNI_T1_1mm template and the FA skeleton (green). Red-Yellow voxels represent regions in which AD was increased significantly in Tourette syndrome patients relative to healthy controls. Axial slices from Z = -20 to 38 in MNI coordinate are shown.

**Figure 3:**
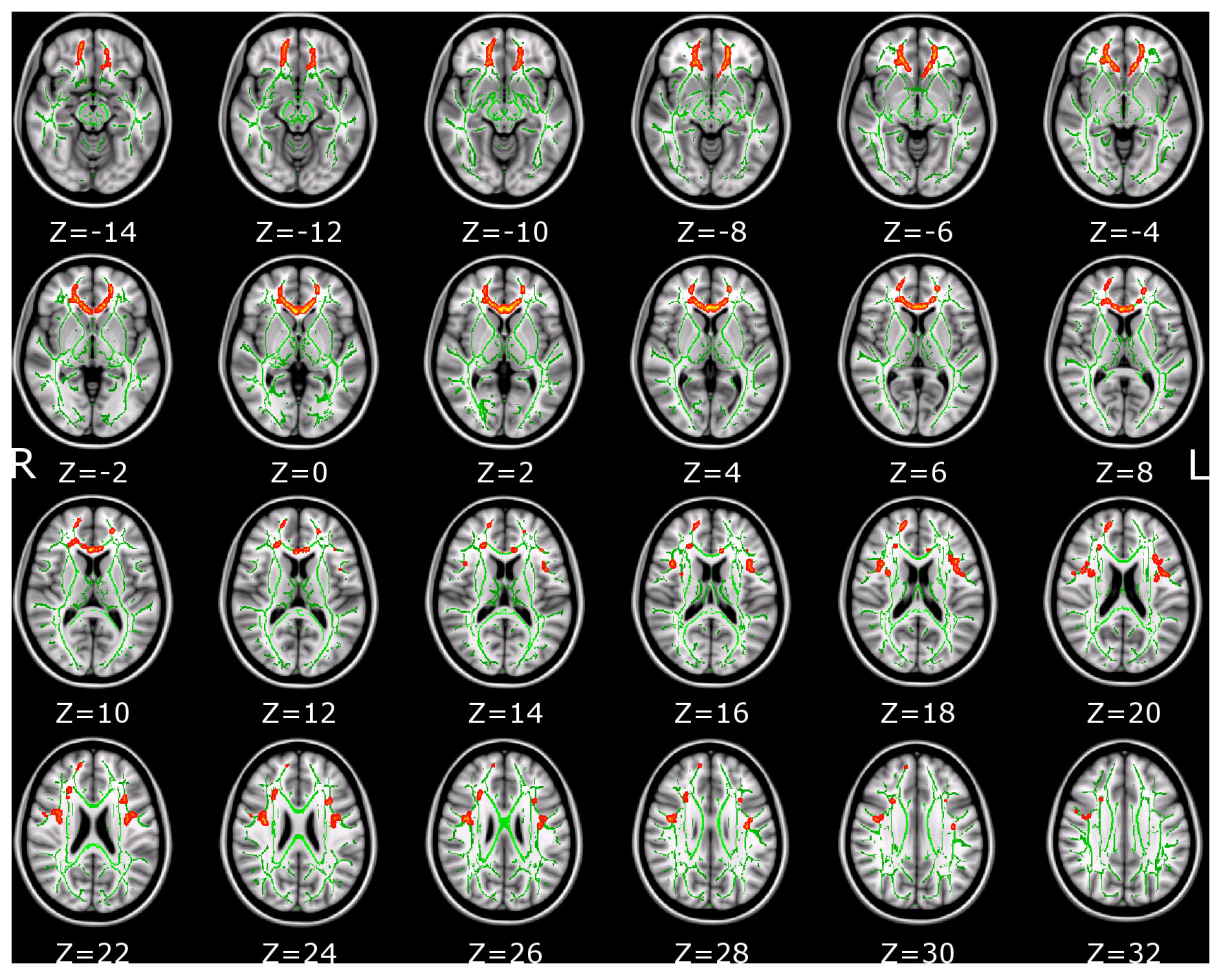
White matter structures showing significantly increased MD in Tourette syndrome patients (corrected for multiple comparisons, p < 0.05). Filled using tbss_fill script of FSL to aid visualization. The background image is the standard MNI_T1_1mm template and the FA skeleton (green). Red-Yellow voxels represent regions in which MD was increased significant in Tourette syndrome patients relative to healthy controls. Axial slices from Z = -14 to 32 in MNI coordinate are shown.

Patients with Tourette syndrome showed significant AD increase in multiple white matter tracts, including anterior thalamic radiation, corticospinal tract, inferior fronto-occipital fasciculus, inferior longitudinal fasciculus and uncinate fasciculus. The coordinates of the local maxima and cluster size are listed in Table 3. Increases of AD are displayed in Figure 2. Significant changes of MD were detected but not for RD. Locations with increased MD are shown in Table 4 and Figure 3. To aid visualization, regions showing significant AD and MD changes (p < 0.05, corrected for multiple comparisons) are thickened using the *tbss_fill* script implemented in FSL (*http://www.fmrib.ox.ac.uk/fsl/tbss/index.html*).

**Table 3:** Skeleton clusters showing significantly increased AD at *p* < 0.05.

Tracts	Voxels	p-value	MNI coordinates (mm)		
			X	Y	z
*Right anterior thalamic radiation*	30505	0.001	9	-21	-15
*Left anterior thalamic radiation*	5571	0.005	-11	-53	-25
*Right Inferior longitudinal fasciculus*	257	0.047	39	3	-28
	37	0.050	51	-20	2
	12	0.050	53	-18	1

**Table 4:** Skeleton clusters showing significantly increased MD at *p* < 0.05.

Tracts	Voxels	p-value	MNI coordinates (mm)				
			X	Y		z	
*Forceps minor*	1417	0.043	1	29		5	
	227	0.048	-15	38		-11	
*Right superior longitudinal fasciculus*	165	0.048	38	-3		24	
	60	0.049	41	14		16	
	22	0.049	51	2		34	
*Left superior longitudinal fasciculus (temporal part)*	93	0.049	-41	3		20	
*Left superior longitudinal fasciculus*	91	0.049	-39	13		16	
	76	0.049	-35	-3		21	

**Figure 4:**
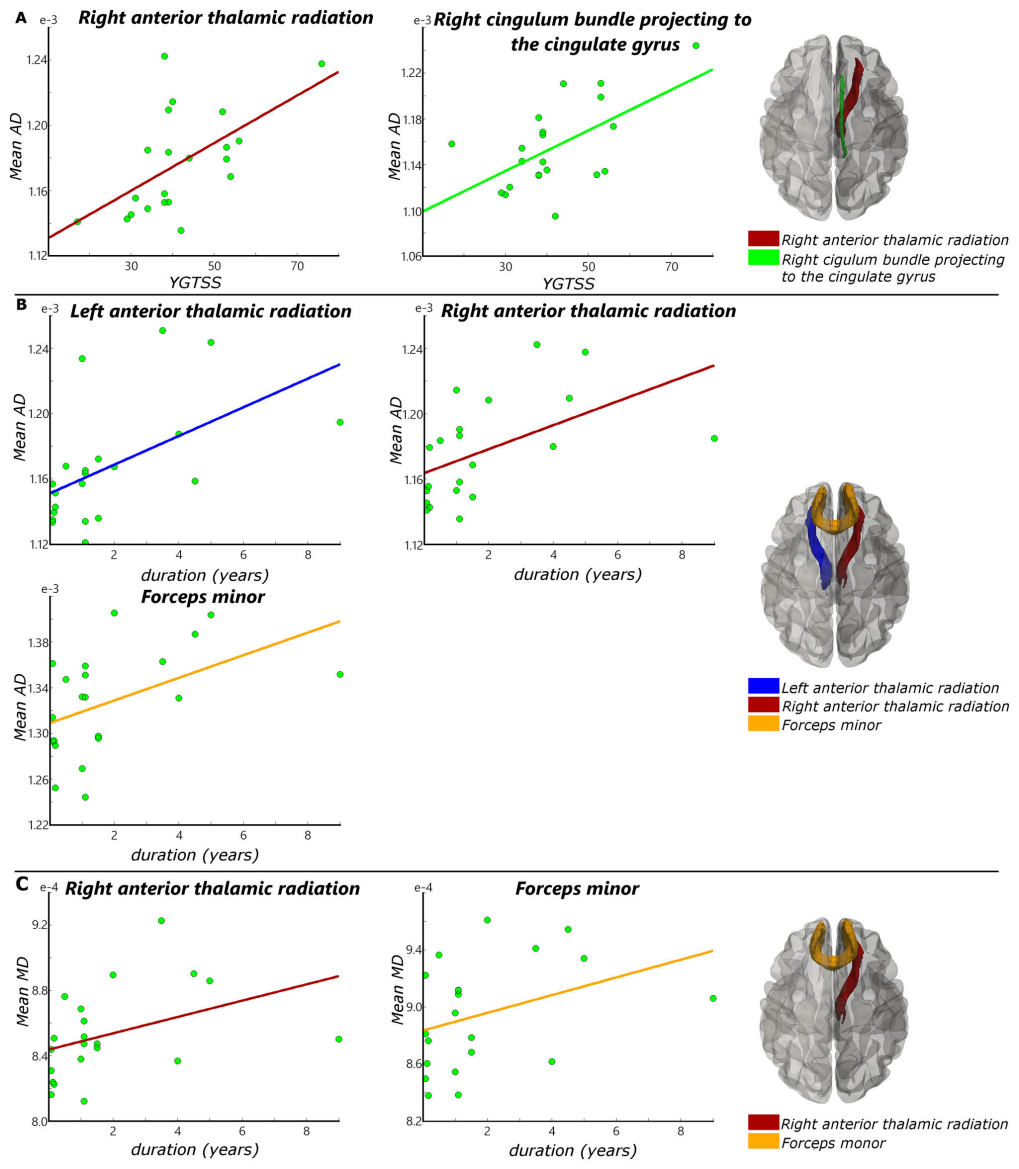
Correlation analysis results of DTI. (A) Significant correlation between YGTSS and mean AD value (Right anterior thalamic radiation: r = 0.531, p = 0.019. Right cingulum bundle projecting to the cingulate gyrus: r = 0.546, p = 0.016). (B) Significant correlation between tics duration and mean AD value (left anterior thalamic radiation: r = 0.554, p = 0.014. Right anterior thalamic radiation: r = 0.584, p = 0.007. Forceps minor: r = 0.682, p = 0.001). (C) Significant correlation between tics duration and mean MD value (Right anterior thalamic radiation: r = 0.508, p = 0.026. Forceps minor: r = 0.593, p = 0.007).

To learn about the functional relevance of the changes, we correlated volumetric and microstructural measures with two functional parameters: tic severity score (YGTSS) and tics duration. After controlling for age, gender, and the whole brain volume, only WMV in the right frontal pole was significantly correlated (r = -0.611, p = 0.005; Figure 1C) with YGTSS scores of the TS children group, showing that severity of impairment was associated with smaller WMV. No other correlations between volume change and YGTSS scores or tic duration were found. AD value in right anterior thalamic radiation (r = 0.531, p = 0.019) and right cingulum bundle projecting to the cingulate gyrus (r = 0.546, p = 0.016) were positively correlated with YGTSS scores of the patient group, when controlled for age and gender (Figure 4A). We did not find any significant correlation between MD values and YGTSS scores. AD values in left anterior thalamic radiation (r = 0.554, p = 0.014), right anterior thalamic radiation (r = 0.584, p = 0.007), and forceps minor (r = 0.682, p = 0.001) had significant positive correlation with patients’ duration of tics (Figure 4B). MD value in right anterior thalamic radiation (r = 0.508, p = 0.026) and forceps minor (r = 0.593, p = 0.007) were also positively correlated with patients’ duration of tics (Figure 4C).

## Discussion

In this study, we investigate TS children with short disease duration, who had Tourette syndrome without ADHD, OCD, or other disorders. Less complications from other disorders allowed us to minimize confounding factors that may influence brain structure and/or function. To the best of our knowledge, this study is the first to combine VBM and TBSS to detect brain morphological and microstructural changes in TS children. We found widespread structural alterations in TS children brain, including frontal cortices, motor-sensory, and left cingulum bundle projecting to the cingulate gyrus. TS children showed not only regional GMV increase and WMV decrease in somatosensory cortex (BA3) and primary motor cortex (BA4), but also presented with AD and MD increase in corticospinal tract, left cingulum bundle projecting to the cingulate gyrus and forceps minor. Furthermore, we noted widespread changes (increased AD) in the anterior limb of the internal capsule. Overall our results indicate that TS is not restricted to motor pathways, but also affects association fibers such as the inferior fronto-occipital fascicle, the superior/inferior longitudinal fascicle and uncinate fascicle as well.

### Alterations in motor-sensory areas, CSTC and limbic structures

We found increased GMV and decreased WMV in the motor-sensory gyrus. This indicates that the main motor pathway—the corticospinal tract—is affected by TS. This, in turn, may have some relation to the conclusion that prefrontal cortical regions, sensory-motor pathways, and limbic structures take part in the modulation of tics [37]. AD values in right anterior thalamic radiation and cingulum bundle projecting to the cingulate gyrus were positively correlated with YGTSS scores. That is, more severe tics are corresponding to higher AD values or larger AD increase. The AD increase starts from the corona radiata and extends to the anterior thalamic radiation, conforming the results of WMV decrease in these areas. The CSTC circuits are believed to be connected directly to the ventral striatum where motivational and incentive behaviors are regulated [38]. The anterior thalamic radiation connects the medial thalamic nuclei and the prefrontal lobe and the cingulum bundle, which in turn, is of key importance in the tics’ pathophysiologic mechanism [39-41]. The AD increase in anterior thalamic radiation indicates the micro-structural changes in the CSTC. Forceps minor of corpus callosum takes part in the modulation of attention by transferring information between the hemispheres [42] and inhibition of motor cortical activities [43]. The AD and MD increase in the forceps minor may result from reduced cell density [9,44,45], indicating reduced interhemispheric connectivity via the corpus callosum, hence reduced interhemispheric inhibition or less long-range interaction control of the motor system.

### Alterations suggesting regulation of tics symptoms

Tics are the hallmark of TS. It is known that interactions of the frontal cortex and striatum guide and regulate motor behavior [46,47]. Considerable evidence suggests that the cortical portion of these connections is involved in regulating the severity of tic symptoms. Regional volumes of the dorsal prefrontal and parietal cortex were found to be significantly larger in children and adults with TS compared with healthy controls [12]. In our research, the decrease of WMV in right superior frontal gyrus was inversely associated with YGTSS scores (r = -0.594, p = 0.009). TS patients with the smaller WMV displayed more tics. Previous brain morphological study results obtained in TS children are different from those obtained in adults [11,48]. Regional volumes of the dorsal prefrontal and parietal cortex were found to be larger in TS children than the controls, whereas it was significantly smaller in TS adults compared to the controls. The volume enlargement was seen as sign of compensatory function. Morphometry study results of other areas like hippocampus, amygdale, and corpus callosum were inconsistent, even when the subjects were all children [48]. This may have resulted from comorbid diseases and/or secondary impairments, which we were able to avoid. We reduced the probability of confounds by studying short disease duration TS children.

### Cross-validation of morphological and microstructural changes

Using DTI we found that the white matter was wildly influenced in our TS children. WMV decrease cluster size as measured by VBM study was about ten times greater than the GMV decrease, and more WM than GM areas showed volume decrease. Furthermore, the only correlation of volume change with tic severity score (YGTSS) was WMV decrease in right frontal pole. Our results showed decreased WMV in frontal lobe of TS children which was consistent with Kates et al [19], but conflicted with Frederickson et al [18]. This discrepancy may have resulted from the difference in segmentation method and patients’ tic duration.

Our comprehensive analysis of brain white matter alterations in TS children demonstrates signiﬁcant WM volume loss in different structures such as somatosensory cortex (BA3), primary motor cortex (BA4), frontal pole and fusiform gyrus, which contains corticospinal tract, left cingulum bundle and forceps minor projections from the frontal-parietal-temporal areas. Forceps minor is a WM pathway that connects the lateral and medial areas of the prefrontal cortex. The WM volume reduction along these fiber pathways was much greater than the grey matter loss in our study. When viewed in conjunction with the adult studies which reported mainly loss of grey matter [14,25], this suggests a difference in change pattern between TS children and adults. This conclusion is consistent with previous reports showing increased AD in the corticospinal tract and post- and pre-central gyrus found in TS adults [15,17]. No significant FA decrease was found. This may indicate AD and MD are more sensitive to microstructural changes in TS children than other DTI indices.

### Pathological reasons and brain development of TS children

Evidences suggested that Tourette syndrome was caused by genetic variations [49,50]. Some children thus do not produce enough myelin, or they have metabolic problems that may cause white matter degeneration. Because cerebral WM plays a fundamental role in transmitting electrical signals between neurons both in terms of short and long-range connections in the brain, it may be that such white matter problems become only apparent (symptomatic) when the TS child matures. The lack of activation input that follows white matter loss or the morphological loss of axons may lead to both anterograde and retrograde degeneration of brain (“use it or lose it”), which also prevents normal GM function. The associated social isolation and the suppression of the symptoms by the TS child may make the tics even more severe, which would then be a non-developmental, secondary problem. Brain development during childhood is a complex and dynamic process for which the pathological mechanism of TS can be quite complicated.

### Limitations

Despite these interesting findings, our study had some limitations. The number of our participants was too small to allow generalization of the radiological findings to other patients with TS. The TS severity score has a large range and so does the standard deviation of the correlations. Also, to save the scanning time, we applied 5mm slice thickness in DTI, which is not the preferred method. In addition, we did not expand our study to follow through to patients’ adulthood, making it difficult to answer why many TS patients improves as they enter adulthood.

## Conclusion

Compared to normal control subjects, the TS children showed a special pattern of WMV decrease, which inversely related to tic severity, and AD/MD increase of subcortical areas underneath the left primary motor-sensory pathway and cingulum bundle projecting to the hippocampus. The increase of GMV may represent an adaptive response of the sensory-motor system and hippocampus to primary pathologic neural input or processing in TS, which possibly allowed partial compensation of abnormal behavior. Increases of AD and MD can be seen as a sign of reduced interhemispheric connectivity, hence reduced interhemispheric inhibition or less long-range interaction control of the motor system.
